# Genome statistics and phylogenetic reconstructions for Southern Hemisphere whelks (Gastropoda: Buccinulidae)

**DOI:** 10.1016/j.dib.2017.11.021

**Published:** 2017-11-07

**Authors:** Felix Vaux, Simon F.K. Hills, Bruce A. Marshall, Steve A. Trewick, Mary Morgan-Richards

**Affiliations:** a Ecology Group, Institute of Agriculture and Environment, Massey University, Palmerston North, New Zealand; b Museum of New Zealand Te Papa Tongarewa, Wellington, New Zealand

## Abstract

This data article provides genome statistics, phylogenetic networks and trees for a phylogenetic study of Southern Hemisphere Buccinulidae marine snails [1]. We present alternative phylogenetic reconstructions using mitochondrial genomic and 45S nuclear ribosomal cassette DNA sequence data, as well as trees based on short-length DNA sequence data. We also investigate the proportion of variable sites per sequence length for a set of mitochondrial and nuclear ribosomal genes, in order to examine the phylogenetic information provided by different DNA markers. Sequence alignment files used for phylogenetic reconstructions in the main text and this article are provided here.

**Specifications Table**

**Table:** 

Subject area	Biology
More specific subject area	Phylogenetics; Genetics; Evolutionary Biology
Type of data	Table, text file, graph, figure
How data was acquired	High-throughput and Sanger DNA sequencing
Data format	Text file format for DNA sequence alignments and phylogenetic trees is.nex (nexus) and.tree respectively.
Experimental factors	Total DNA was extracted from specimens using CTAB buffer. DNA was paired-end sequenced using the high-throughput Illumina HiSeq. 2500 platform. Short-length DNA sequences were amplified via PCR and Sanger sequenced.
Experimental features	mtDNA genome and 45 S nuclear ribosomal DNA sequences were assembled using reference sequences. Sequences were aligned with gaps and poorly-aligned positions removed. Phylogenetic trees were constructed using Bayesian (BEAST 1.8.3) and Maximum-Likelihood methods (RAxML 8.2.8). The unrooted phylogenetic network of some alignments was investigated using SplitsTree 4.
Data source location	Most specimens originate from New Zealand waters, some were collected from the coasts of Australia, Japan, USA (California), and the UK.
Data accessibility	Interactive.nwk (Newick) tree files are provided here and with the main article [1].

**Value of the data**

• Summary statistics for whole mitochondrial DNA sequences and 45S nuclear ribosomal genes are presented because such information for gastropods is currently rare, and base bias is known to influence phylogenetic inferences.
• Phylogenetic reconstructions (from short-length DNA sequence data) presented here include multiple buccinid and buccinulid taxa not included in the main-text trees, and may be useful to future evolutionary studies of Neogastropoda.
• DNA sequence variation and phylogenetic trees are provided because Southern Hemisphere taxa are currently under-sampled.
• The proportion of variable DNA sites for a selection of mitochondrial and nuclear genes from buccinulid whelks are compared. This information can improve genetic marker selection for future molluscan studies.

## 1 Data

The data presented here originates from a phylogenetic study of Southern Hemisphere whelks [1], which refers to a group of marine snails that can be classified within the taxonomic families Buccinulidae or Buccinidae [2], [3], [4], [5], [6], [7], [8]. The classification of these gastropod snails depends upon a biogeographic hypothesis and an assumption of reciprocal monophyly between the majority of lineages in the Northern and Southern Hemispheres [3], [6], [7], [8]. Results from our study indicated that Buccinulidae and Southern Hemisphere whelks are not monophyletic [1].

32 putative buccinulid and buccinid marine snails, as well as three fasciolariid snails used as a phylogenetic outgroup, were high-throughput sequenced on the Illumina 2500 platform. Sequence data was assembled to provide mitochondrial (mtDNA) genomic and 45S nuclear ribosomal DNA (rDNA) sequence data for most taxa, although some individuals failed to successfully sequence for the entire mtDNA or rDNA. This data was complemented with short-length sequence data from the mitochondrial 16S RNA and *cox1* genes and nuclear ribosomal 28S RNA gene. This short-length sequence data was acquired via PCR amplification and Sanger sequencing using universal primers. Sequence alignments used for analyses presented in the main text are attached to this paper.

**Fig. 1:**
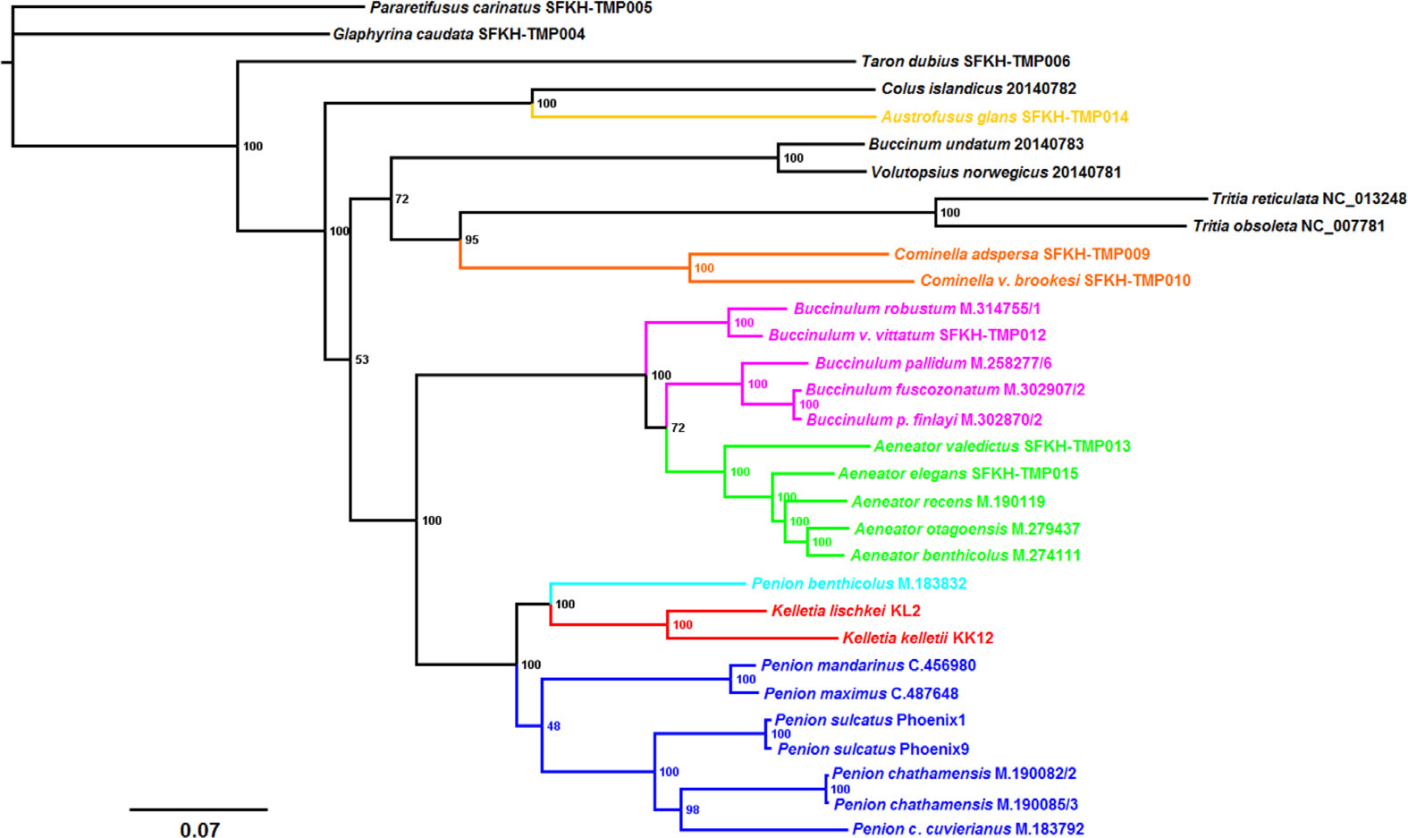
*Maximum-likelihood mtDNA phylogeny of buccinid and buccinulid whelks*. A maximum-likelihood derived phylogeny generated using RAxML 8.2.8 [9], based an alignment of 31 concatenated mitochondrial genome sequences (11,128 bp incorporating protein-encoding, tRNA and rRNA genes). No partitions were used. No outgroup or monophyly was enforced for this tree. Genera putatively belonging to Buccinulidae are shown in different colours.

Using these sequence alignments, we present maximum-likelihood and Bayesian phylogenetic reconstructions for the sampled buccinulid whelks. These phylogenetic trees are alternative reconstructions that can be compared to trees presented in the main text. Splits networks are also estimated using the mtDNA genomic and nuclear ribosomal RNA (18S, 5.4S, 28S) sequence data. The proportion of variable sites per sequence length for a set of mitochondrial and nuclear ribosomal genes is investigated as well, which provides insight towards marker information for recent and distant evolutionary change in neogastropods (Fig. 1, Fig. 9).

**Fig. 2:**
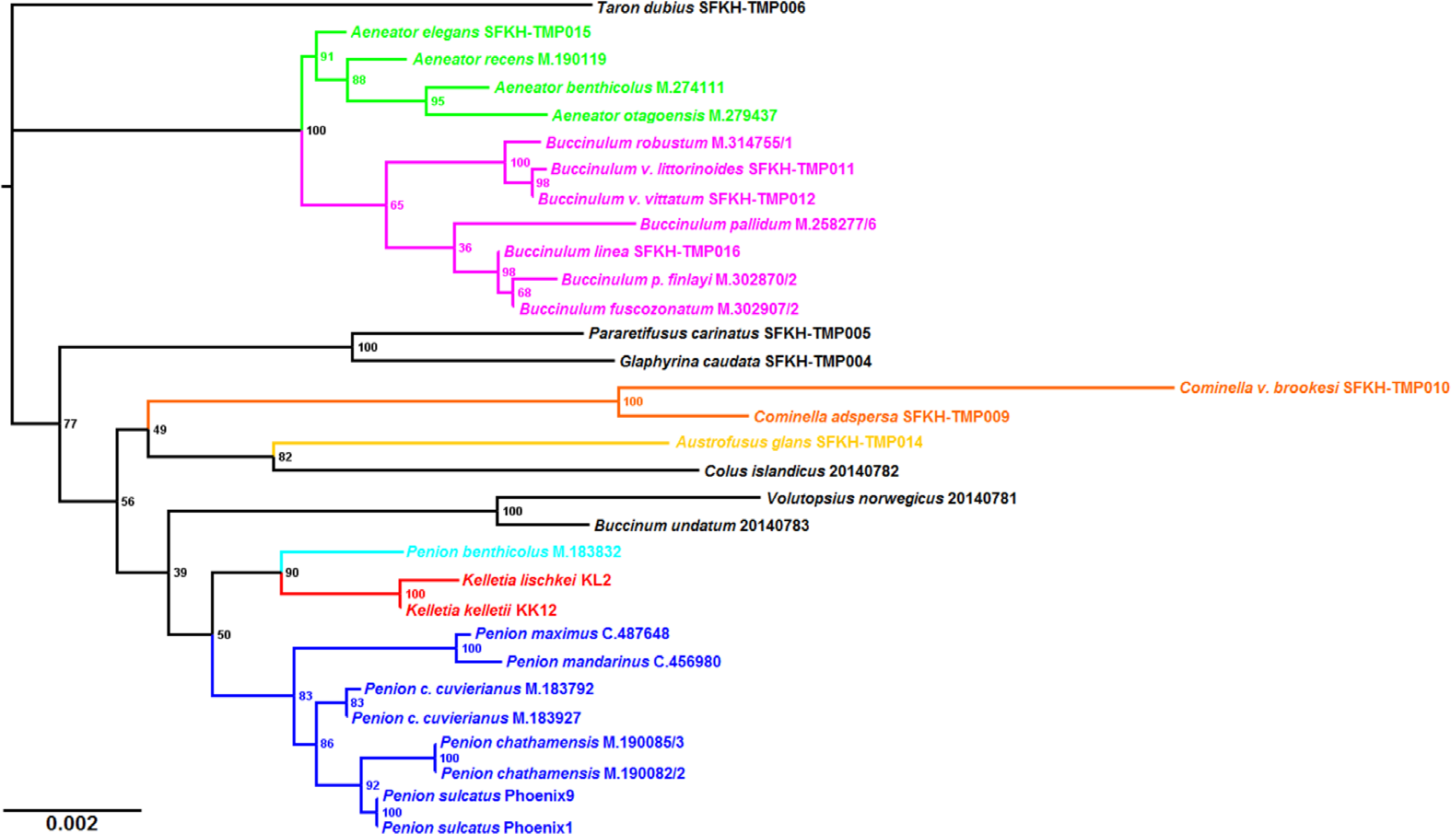
*Maximum-likelihood 45S rDNA phylogeny of buccinid and buccinulid whelks*. A maximum-likelihood derived phylogeny generated using RAxML 8.2.8 [9], based on a 4667 bp alignment of 31 concatenated nuclear rDNA gene sequences (18S, 5.8S, 28S rRNA). No partitions were used. No outgroup or monophyly was enforced for this tree. Genera putatively belonging to Buccinulidae are shown in different colours.

**Fig. 3:**
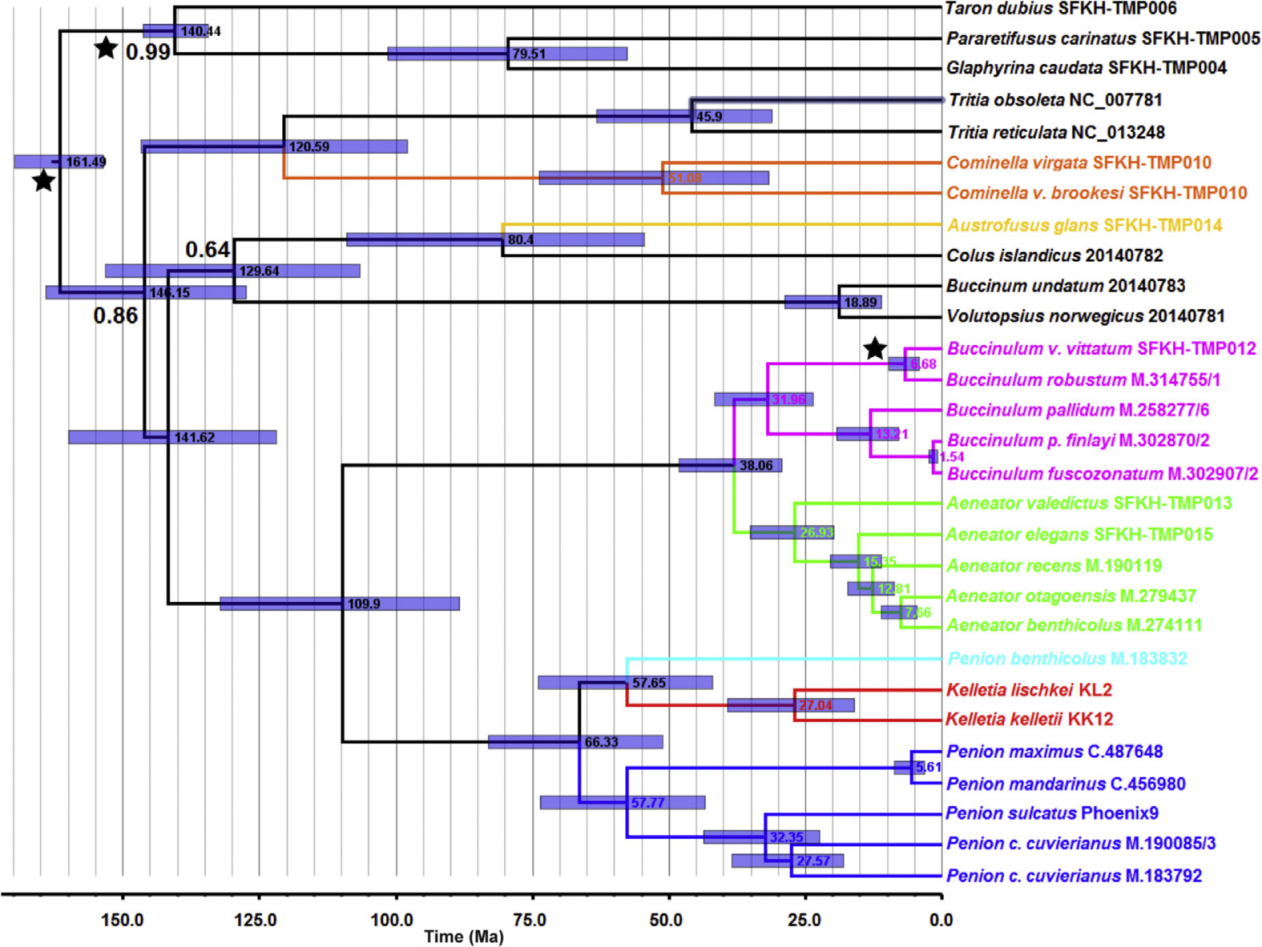
*Bayesian calibrated mtDNA phylogeny of buccinid and buccinulid whelks*. A Bayesian phylogeny based on an alignment of 25 concatenated mitochondrial genome sequences (incorporating protein-encoding, tRNA and rRNA genes), which has been fossil calibrated to estimate divergence dates among the whelk lineages. Two sequence partitions were used: 1) protein-encoding and tRNA genes (10,635 bp), and 2) tRNA genes (1065 bp) using the GTR + I + G and HKY + I + G substitution models respectively [10], [11]. Black stars indicate splits that fossil calibrated. Tree root height was calibrated using the earliest known buccinoid fossils [12], and fossil calibrations were also used for the earliest Fasciolariidae (un-enforced outgroup) [13], [14], and the earliest known occurrence of the tip branch *Buccinulum v. vittatum*[15]. BEAST 1.8.3 [16] using and MCMC length of 100 million, 1000 sample frequency and a 10% burn-in was used to generate this phylogeny. Node labels are estimated median divergence dates with the 95% highest posterior density (HPD) range shown as a blue bar. Posterior support values are also shown at nodes, but only if support was < 1.0. Putative buccinulid genera are shown in different colours.

**Fig. 4:**
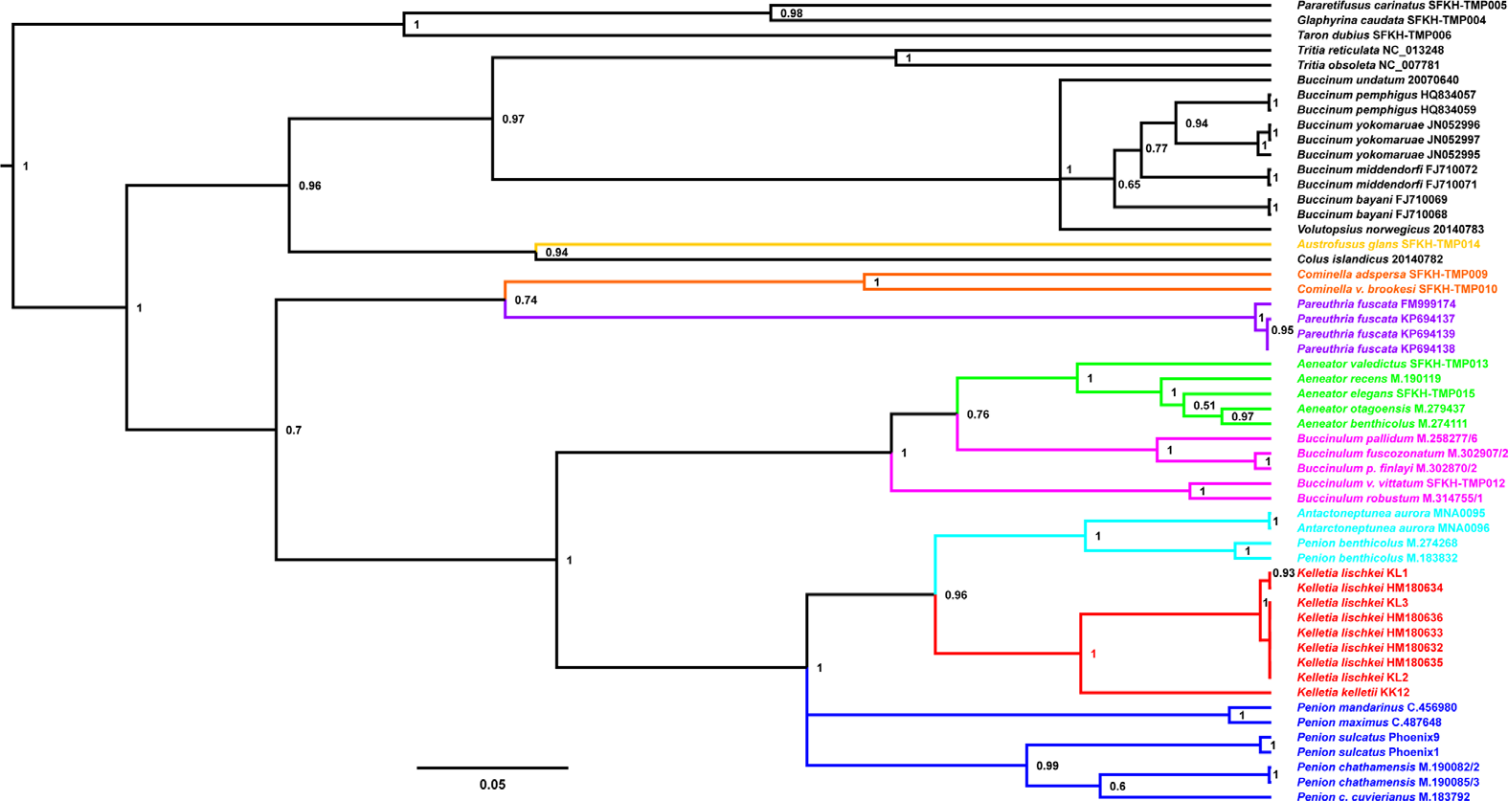
*Bayesian cox1 phylogeny of buccinid and buccinulid whelks*. A Bayesian phylogeny based on a 439 bp alignment of mitochondrial *cox1* gene sequences obtained from 54 individual marine snails. The GTR + I + G substitution model was used [11]. The phylogeny was produced using a Bayesian method (100 million MCMC, 10% burn-in, 1000 sample frequency, node labels are posterior support values), via BEAST 1.8.3 [16]. For this tree no outgroup was specified explicitly but reciprocal monophyly was enforced for the Fasciolariidae and Buccinidae/Buccinulidae/Nassariidae. Genera putatively belonging to Buccinulidae are shown in different colours.

**Fig. 5:**
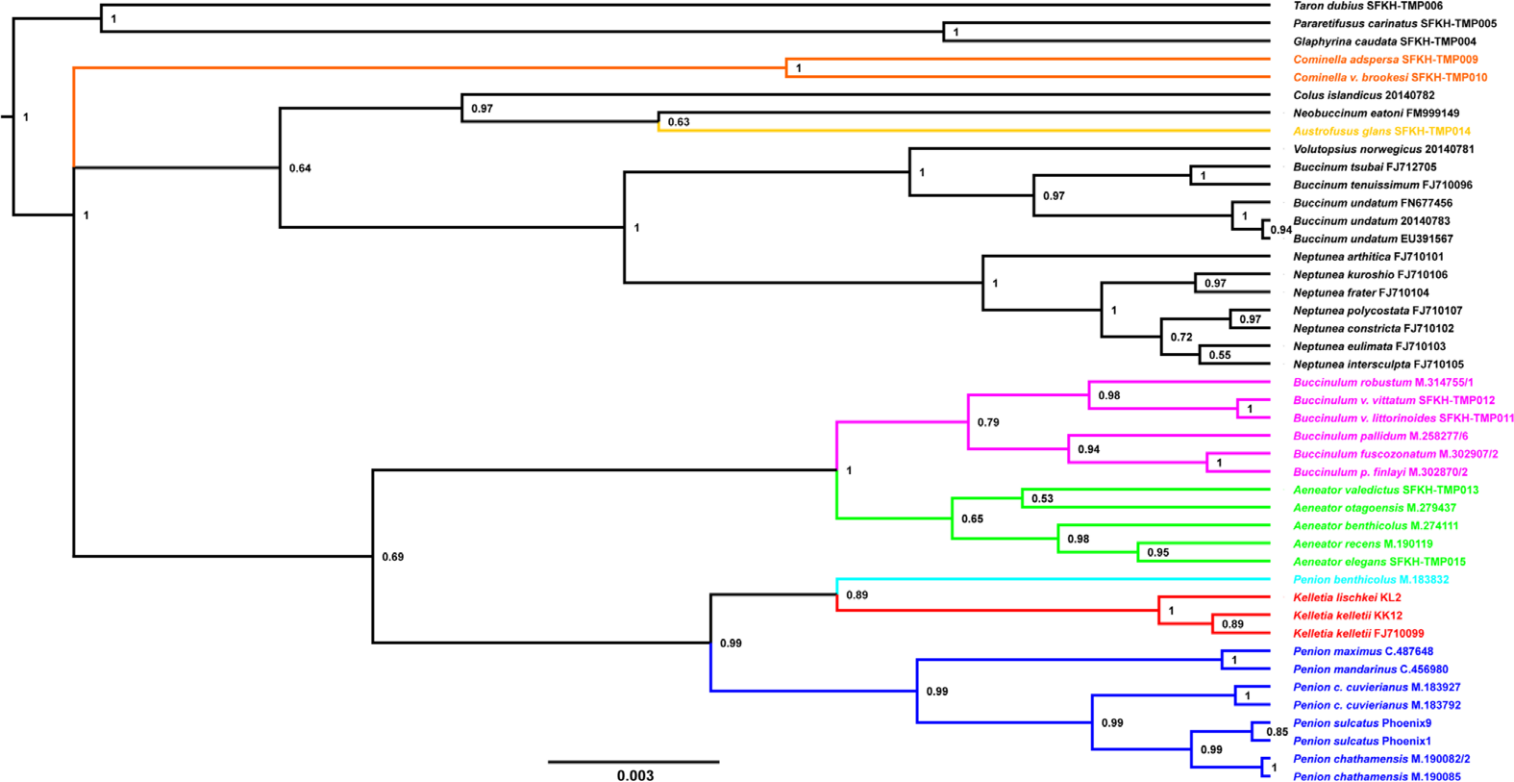
*Bayesian 28S RNA phylogeny of buccinid and buccinulid whelks*. A Bayesian phylogeny based on an alignment of nuclear ribosomal 28 S RNA gene sequences obtained from 44 individual marine snails (1486 bp). The GTR + I + G substitution model was used [11]. The phylogeny was produced using a Bayesian method (100 million MCMC, 10% burn-in, 1000 sample frequency, node labels are posterior support values), via BEAST 1.8.3 [16]. For this tree no outgroup was specified explicitly but reciprocal monophyly was enforced for the Fasciolariidae and Buccinidae/Buccinulidae/Nassariidae. Genera putatively belonging to Buccinulidae are shown in different colours.

**Fig. 6:**
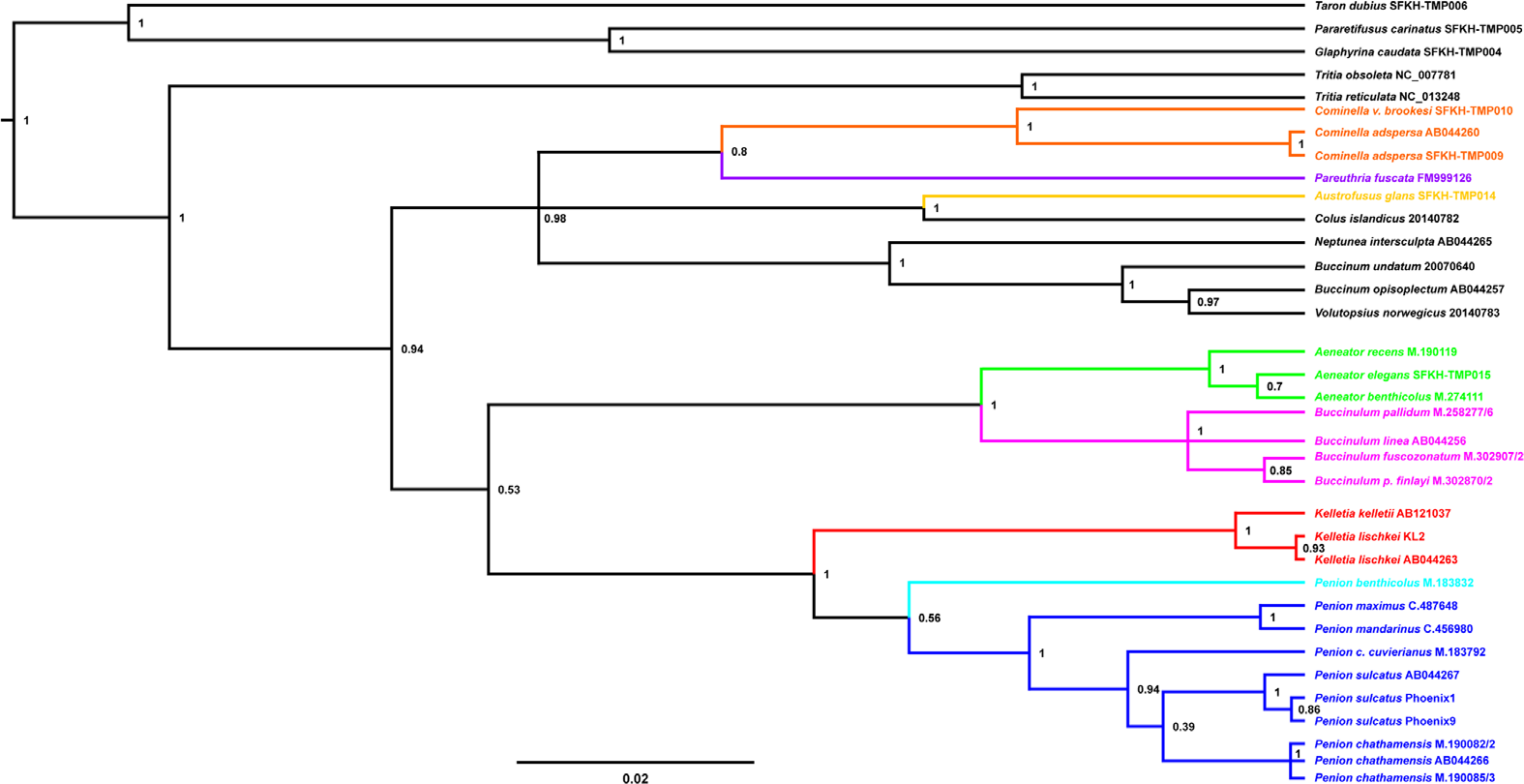
*Bayesian 16S RNA phylogeny of buccinid and buccinulid whelks*. A Bayesian phylogeny based on an alignment of mitochondrial 16S RNA gene sequences obtained from 35 individual marine snails (868 bp). The GTR + I + G substitution model was used [11]. The phylogeny was produced using a Bayesian method (100 million MCMC, 10% burn-in, 1000 sample frequency, node labels are posterior support values), via BEAST 1.8.3 [16]. For this tree no outgroup was specified explicitly but reciprocal monophyly was enforced for the Fasciolariidae and Buccinidae/Buccinulidae/Nassariidae. Genera putatively belonging to Buccinulidae are shown in different colours.

**Fig. 7:**
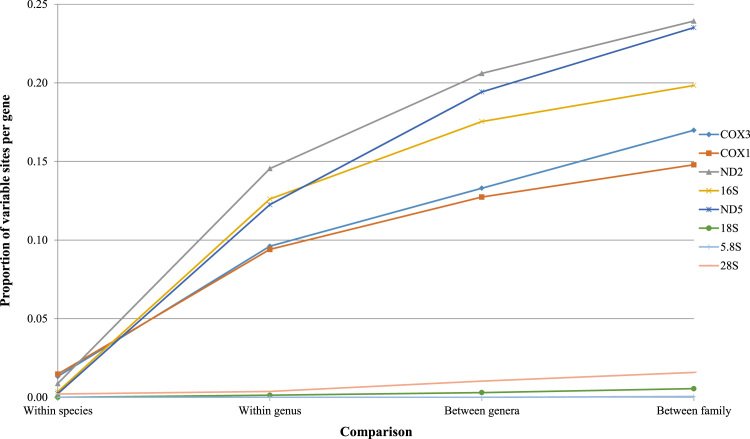
*Proportion of variable sites at increasingly deep levels of divergence*. The proportion of variable sites per sequence length (bp) for a selection of mtDNA and nuclear rDNA genes reflects different rates of DNA substitution. Values were calculated using Geneious 9.1.3 [17]. The trends plotted effectively represent change in the phylogenetic information provided by each gene for different levels of investigation. Average numbers of variable sites were used for groups in genus and family-level comparisons. For example, we used the average number of differences for all sampled whelk (Buccinidae/Buccinulidae) taxa from all sampled Fasciolariidae taxa. Sampling from *Aeneator*, *Buccinulum* and *Penion* was used to estimate generic-level differences as these groups contained more than two specimens. Likewise, only *P. sulcatus, P. chathamensis*, and *P. c. cuvierianus* were used for within-species estimates as these taxa were sampled twice. Since read coverage varies for some genes, not all individuals were included for estimates made for each gene.

**Fig. 8:**
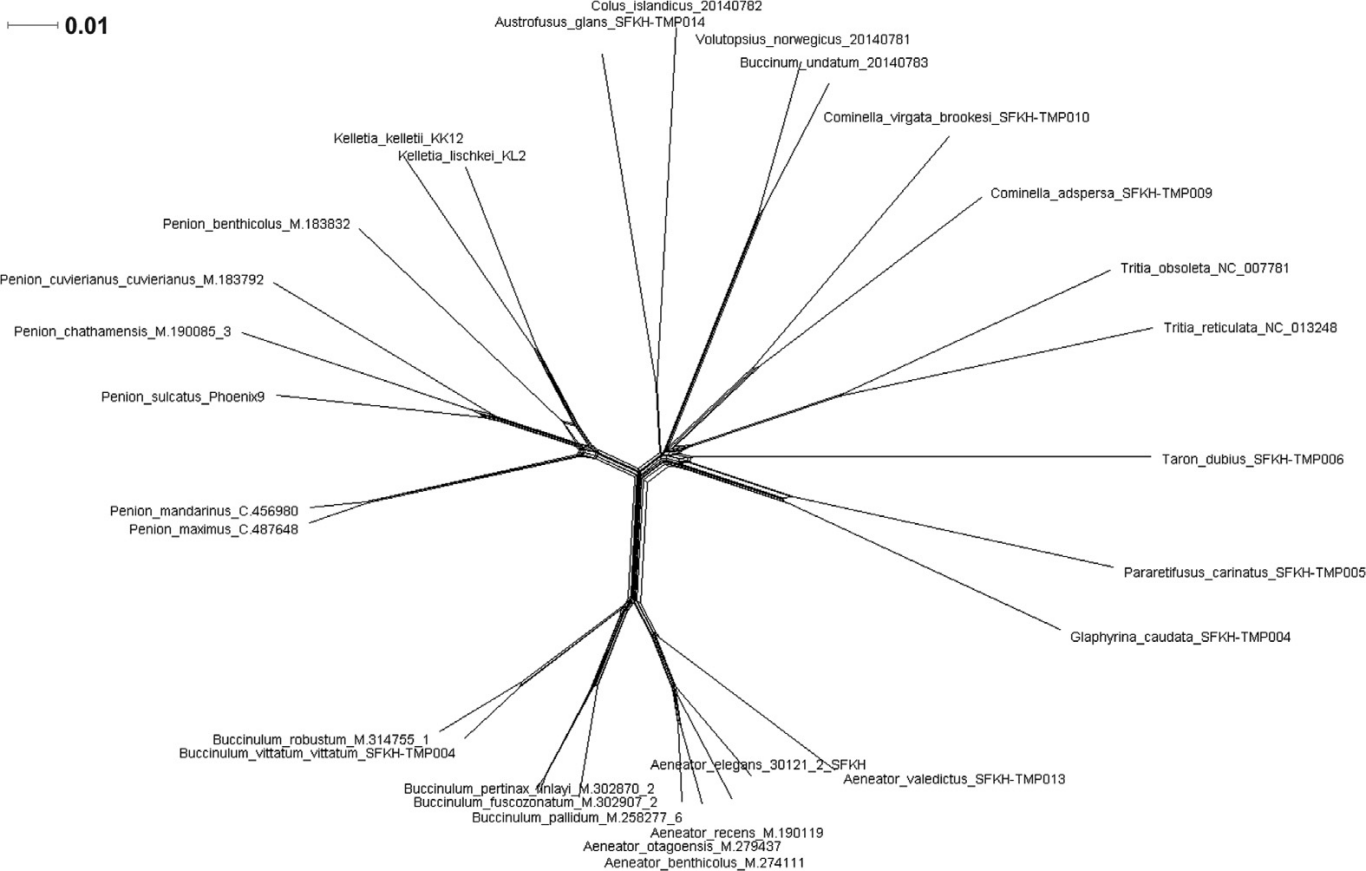
*Splits network illustrating alternative phylogenetic signal in mtDNA sequence data for marine snails*. The splits network of based on an alignment of 31 concatenated mitochondrial genome sequences (incorporating protein-encoding, tRNA and rRNA genes; 11,128 bp). Splits were generated using the Neighbor-Net algorithm in SplitsTree 4 [18]. The splits network presents a generalisation of all of possible topological solutions for the phylogenetic signal contained in the underlying sequence data, but it does not quantify the likelihood of alternative phylogenetic relationships. Edge length is proportional to split weight, and box structures within the network indicate signal for alternative topologies in the underlying sequence data.

**Fig. 9:**
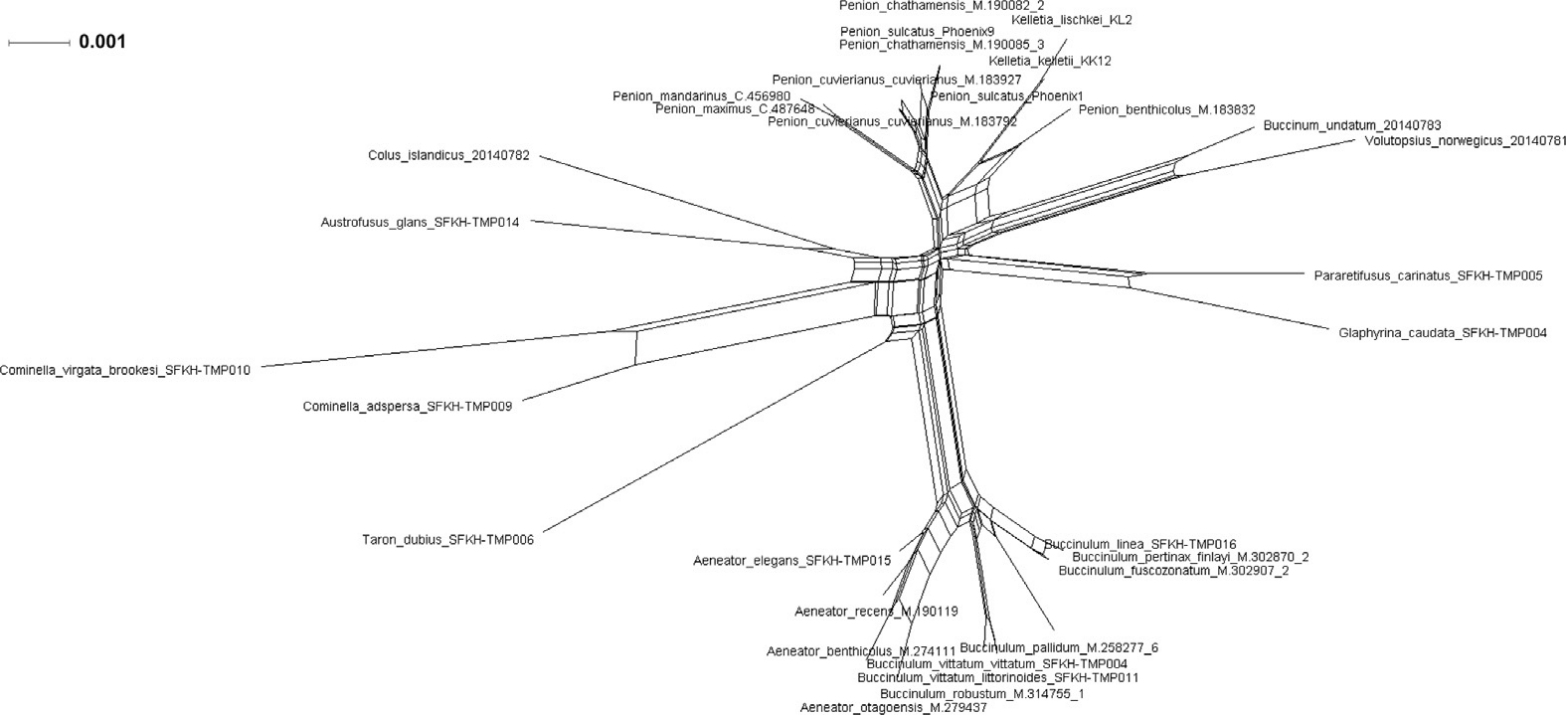
*Splits network for illustrating alternative phylogenetic signal in 45S rDNA sequence data for marine snails*. The splits network of based on a 4667 bp alignment of 31 concatenated nuclear rDNA gene sequences (18S, 5.8S, 28S rRNA genes). Splits were generated using the Neighbor-Net algorithm in SplitsTree 4 [18]. The splits network presents a generalisation of all of possible topological solutions for the phylogenetic signal contained in the underlying sequence data, but it does not quantify the likelihood of alternative phylogenetic relationships. Edge length is proportional to split weight, and box structures within the network indicate signal for alternative topologies in the underlying sequence data.

## 2 Experimental design, materials and methods

The DNA extraction, purification, sequencing method and routine for sequence assembly is provided in the main text [1]. The main text also explains how the figures presented here were generated, including the software and settings used. Legends for tables and figures presented below specify which sequence alignments were used (again referenced in the main text) (Table 1, Table 2).

**Table 1:** A summary of statistics for the length and nucleotide composition for the concatenated DNA sequences for the nuclear ribosomal RNA genes 18S, 5.8S and 28S (the internal transcribed spacer regions are not included). All listed specimens were newly sequenced for this study.

**Species**	**Museum ID**	**Concatenated nuclear rDNA 18S, 5.8S, 28S**					
		**Length (bp)**	**% A**	**% C**	**% G**	**% T**	**GC bias**
*Pararetifusus carinatus*	SFKH-TMP005	5337	23	24.5	30.0	22.2	54.5
*Glaphyrina caudata*	SFKH-TMP004	5339	23	24.5	30.0	22.2	54.5
*Taron dubius*	SFKH-TMP006	5339	23	24.7	30.1	22.0	54.8
*Austrofusus glans*	SFKH-TMP014	5338	24	24.4	30.0	22.2	54.4
*Colus islandicus*	20140782	5334	24	24.5	30.0	22.2	54.5
*Volutopsius norwegicus*	20140781	5338	24	24.4	30.0	22.3	54.4
*Buccinum undatum*	20140783	5339	24	24.3	30.0	22.3	54.3
*Cominella adspersa*	SFKH-TMP009	5339	23	24.6	30.0	22.0	54.6
*Cominella v. brookesi*	SFKH-TMP010	5339	21	24.9	30.3	21.7	55.2
*Buccinulum fuscozonatum*	M.302907/2	5340	22	24.8	30.1	22.0	54.9
*Buccinulum linea*	SFKH-TMP016	5340	22	24.8	30.1	22.0	54.9
*Buccinulum v. littorinoides*	SFKH-TMP011	5340	22	24.7	30.1	22.0	54.8
*Buccinulum pallidum*	M.258277/6	5340	22	24.7	30.2	21.9	54.9
*Buccinulum p. finlayi*	M.302870/2	5340	22	24.7	30.1	22.0	54.8
*Buccinulum robustum*	M.314755/1	5340	22	24.8	30.1	21.9	54.9
*Buccinulum v. vittatum*	SFKH-TMP004	5340	22	24.7	30.1	22.0	54.8
*Aeneator benthicolus*	M.274111	5340	22	24.6	30.1	22.1	54.7
*Aeneator elegans*	SFKH-TMP015	5340	22	24.7	30.1	22.0	54.8
*Aeneator otagoensis*	M.279437	5340	22	24.7	30.2	21.9	54.9
*Aeneator recens*	M.190119	5340	22	24.6	30.1	22.1	54.7
*Penion benthicolus*	M.183832	5337	23	24.4	30.0	22.3	54.4
*Kelletia kelletii*	KK12	5337	24	24.4	29.9	22.3	54.3
*Kelletia lischkei*	KL2	5337	24	24.3	29.9	22.4	54.2
*Penion mandarinus*	C.456980	5339	24	24.4	29.9	22.2	54.3
*Penion maximus*	C.487648	5339	25	24.4	29.9	22.2	54.3
*Penion sulcatus*	Phoenix9	5339	23	24.4	30.0	22.3	54.4
*Penion sulcatus*	Phoenix1	5339	23	24.4	30.0	22.3	54.4
*Penion chathamensis*	M.190085/3	5339	23	24.4	29.9	22.3	54.3
*Penion chathamensis*	M.190082/2	5339	23	24.4	29.9	22.3	54.3
*Penion c. cuvierianus*	M.183792	5339	23	24.3	30.0	22.3	54.3
*Penion c. cuvierianus*	M.183927	5339	23	24.3	30.0	22.4	54.3

**Table 2:** A summary of the statistics for the length and nucleotide composition for the mitochondrial genomes newly sequenced as part of this study. Specimens marked with one asterisk (*) exhibit drops in read coverage for some small regions, for example *K. kelletii* has 54 bp missing from *cox1*. Specimens marked with two asterisks (**) have genomes with large gaps in genome coverage for some regions, such as *B. v. vittatum* that has 266, 151 and 64 bp missing from the ATP6, *cox1* and ND2 genes respectively.

**Species**	**Museum ID**	**mtDNA genome**						
		**Length (bp)**	**% A**	**% C**	**% G**	**% T**		**GC bias**
*Pararetifusus carinatus*	SFKH-TMP005	15204	31.5	14	15.0	40.1		28.4
*Glaphyrina caudata*	SFKH-TMP004	15235	31.5	13	14.6	40.7		27.9
*Taron dubius*	SFKH-TMP006	15189	29.3	15.7	17.0	38.0		32.7
*Austrofusus glans*	SFKH-TMP014	15195	31.1	14.5	15.3	39.1		29.8
*Colus islandicus*	20140782	15158	30.5	14.9	15.8	38.7		30.7
*Volutopsius norwegicus*	20140781	15232	29.3	15.7	16.5	38.4		32.2
*Buccinum undatum*	20140783	15231	29.5	15.6	16.3	38.7		31.9
*Cominella adspersa*	SFKH-TMP009	15251	30.4	15.7	16.0	38.0		31.7
*Cominella v. brookesi*	SFKH-TMP010	15263	29.6	15.9	16.7	37.8		32.6
*Buccinulum fuscozonatum*	M.302907/2	15246	30.2	14.8	15.8	39.1		30.6
*Buccinulum pallidum*	M.258277/6	15247	30.9	14.1	15.2	39.7		29.3
*Buccinulum p. finlayi*	M.302870/2	15247	30.1	14.8	15.9	39.1		30.7
*Buccinulum robustum*	M.314755/1	15244	29.6	15.2	16.1	39.0		31.3**
*Buccinulum v. vittatum*	SFKH-TMP012	15244	29.6	15.1	16.4	38.9		31.5**
*Aeneator benthicolus*	M.274111	15254	30.4	14.7	15.7	39.2		30.4
*Aeneator elegans*	SFKH-TMP015	15254	30.3	14.6	15.8	39.3		30.4
*Aeneator otagoensis*	M.279437	15249	30.3	14.7	15.5	39.5		30.2*
*Aeneator recens*	M.190119	15264	30.0	14.9	16.0	39.1		30.9
*Aeneator valedictus*	SFKH-TMP013	15258	29.3	15.8	16.7	38.2		32.5
*Penion benthicolus*	M.183832	15229	29.5	16.2	17.0	37.3		32
*Kelletia kelletii*	KK12	15104	29.3	16.0	17.1	37.6		31*
*Kelletia lischkei*	KL2	15225	29.6	16.1	16.8	37.5		32.9
*Penion mandarinus*	C.456980	15250	30.4	15.1	16.2	38.3		31.3
*Penion maximus*	C.487648	15249	30.6	15.1	16.0	38.2		31.1
*Penion sulcatus*	Phoenix9	15227	29.2	16.0	17.2	37.5		32
*Penion sulcatus*	Phoenix1	15227	29.2	16.1	17.2	37.4		33
*Penion chathamensis*	M.190085/3	15227	28.6	16.8	18.0	36.7		34.8
*Penion chathamensis*	M.190082/2	15228	28.5	16.8	18.0	36.7		34.8
*Penion c. cuvierianus*	M.183792	15235	28.6	16.9	17.8	36.7		34.7
*Penion c. cuvierianus*	M.183927	15241	28.3	17.1	18.0	36.6		35.1**
